# Reference intervals for indices derived from the complete blood count in the Longitudinal Study of Adult Health (ELSA-Brasil)

**DOI:** 10.1016/j.htct.2026.108552

**Published:** 2026-10-15

**Authors:** Nívea Aparecida de Almeida, Roberta Carvalho Figueiredo, Sandhi Maria Barreto, Letícia Gonçalves Resende Ferreira, Chams Bicalho Maluf, Pedro Guatimosim Vidigal, Danyelle Romana Alves Rios

**Affiliations:** a Universidade Federal de São João del-Rei, Campus Centro Oeste, Brazil; b Department of Preventive Medicine, School of Medicine, Universidade Federal de Minas Gerais (UFMG) and Hospital das Clínicas/EBSERH, Belo Horizonte, Brazil; c Department of Clinical Pathology, School of Medicine, Universidade Federal de Minas Gerais (UFMG), Belo Horizonte, Brazil

**Keywords:** Biomarkers, Complete blood count, Epidemiology, Reference interval, Systemic inflammation

## Abstract

**Introduction:**

Hematological biomarkers from routine tests are attractive for their accessibility and clinical applicability, particularly indices derived from the complete blood count. Establishing reference intervals is essential for accurate interpretation, requiring healthy populations and standardized methods following Clinical and Laboratory Standards Institute guidelines.

**Objective:**

To establish reference intervals for complete blood count-derived indices in healthy adults.

**Methods:**

This cross-sectional study analyzed baseline data from 2230 participants aged 35–74 years in the Brazilian Longitudinal Study of Adult Health. Individuals with inflammatory conditions or hematological extremes (<1st and >99th percentiles) were excluded. Evaluated cellular parameters comprised five inflammatory ratios (neutrophil-to-lymphocyte, platelet-to-lymphocyte, lymphocyte-to-monocyte, monocyte-to-lymphocyte, and derived neutrophil-to-lymphocyte) and three composite indices (systemic inflammatory response, aggregate index of systemic inflammation, and systemic immune-inflammation). Reference intervals corresponded to the 2.5th–97.5th percentiles. Group differences by sex and age were evaluated using Mann–Whitney and Harris–Boyd tests.

**Results:**

Males constituted 54.3% of the sample, and 92.0% of the participants were aged 35–59 years. Neither sex nor age required data stratification. The established reference intervals were: neutrophil-to-lymphocyte ratio (0.95–3.28), platelet-to-lymphocyte ratio (77.25–213.27), lymphocyte-to-monocyte ratio (2.27–7.17), monocyte-to-lymphocyte ratio (0.14–0.44), derived neutrophil-to-lymphocyte ratio (0.72–2.33), systemic inflammatory response index (0.33–1.89), aggregate index of systemic inflammation (69.85–460.01), and systemic immune-inflammation index (201.64–830.02).

**Conclusion:**

These reference intervals provide a basis for clinical and epidemiological studies on subclinical inflammation and chronic disease risk.

## Introduction

Currently, indices derived from the complete blood count (CBC), calculated from the absolute values of leukocyte subtypes, and platelets, have played a relevant role in inflammatory situations and outcomes [1]. It is known that in the initial phase of the innate immune response, neutrophils are the most abundantly recruited leukocyte subgroup and, when elevated, it appears to indicate a strong association with in-hospital mortality and major adverse cardio- and cerebrovascular events [[2], [3], [4]]. Monocytes and macrophages (derived from monocytes) also contribute to the innate immune response. They are the second subgroup recruited to promote the defense of organisms. When increased, they are associated with a higher risk of adverse cardiovascular events (myocardial injury), atherosclerosis, heart failure, and atrial fibrillation [5,6]. In contrast, a reduction in the lymphocyte count is associated with aggravated inflammation or adverse cardiovascular events [7]. Platelets play a role in the immune system that is as important as their function in primary hemostasis; when increased in peripheral blood, they are associated with a higher cardiovascular risk. Platelets interact with leukocytes, releasing pro-inflammatory factors, such as chemokines and cytokines, contributing to vascular inflammation and modulating the immune response [8].

Due to the inherent variability and instability of individual leukocyte counts during immunoinflammatory responses, ratios derived from these cellular subsets are proposed as more stable biomarkers than absolute values in isolation [4]. Among the complete blood count-derived indices, the most prominent include the neutrophil-to-lymphocyte ratio, platelet-to-lymphocyte ratio, lymphocyte-to-monocyte ratio, monocyte-to-lymphocyte ratio, derived neutrophil-to-lymphocyte ratio, systemic inflammatory response index, aggregate index of systemic inflammation, and systemic immune-inflammation index. These parameters are established markers of systemic inflammation and prognosis in cardiovascular, infectious, and neoplastic conditions [[9], [10], [11], [12]].

Complete blood count-derived indices have been shown to be promising inflammatory markers that assist in evaluating clinical status and prognosis, particularly in cardiovascular and neoplastic diseases [4,13,14]. Studies have shown that changes in these indices are associated with all-cause mortality, long-term mortality after myocardial revascularization, increased risk of developing cardiovascular diseases (CVDs), cancer, and other chronic conditions [[12], [13], [14], [15], [16]].

It is noteworthy that hematological indices can be obtained at a low cost, since the cell counts contained in the CBC, the routine laboratory test most requested by health professionals, are used, thus they are widely available to the population. However, the reference intervals for some of these indices remain undefined. Currently, there is a wide and inconsistent range of cut-off points in the literature, since most of them were obtained from small and restricted populations [17,18]. Reference intervals are necessary for the interpretation and understanding of the index results, and thus, to enhance their usefulness and the clinical validation of the population. The Brazilian Longitudinal Study of Adult Health (ELSA-Brasil), a large prospective cohort, primarily aims to investigate the incidence and determinants of non-communicable chronic diseases [19,20]. The scope of this study encompassed comprehensive laboratory testing, including the CBC, to establish population-specific reference intervals. Given its prospective cohort design, ELSA-Brasil enables a longitudinal evaluation of health outcomes over time and assesses the predictive value of these indices relative to established reference intervals, thereby offering insight into disease trajectories within this population. Therefore, the objective of the present study was to establish reference intervals for indices derived from the CBC in this population.

## Methods

### Study design and population

This study evaluated a subset of participants from the ELSA-Brasil study, a prospective cohort comprising 15,105 adults aged 35–74 years. The participants were active or retired civil servants from higher education or research institutions located in six state capitals of Brazil. Baseline data collection occurred between 2008 and 2010. To date, the study has completed four examination waves: baseline (2008–2010), wave 2 (2012–2014), wave 3 (2017–2019), and wave 4 (2022–2024). Ethics committee authorizations were granted (CAAE 0016.1.198.000–06) and all individuals gave their written informed consent [19,20].

For the present study, of the 15,105 participants in the ELSA-Brasil study, 95 were excluded because CBC data were unavailable. In addition, participants who presented factors that could alter the inflammatory state were excluded (Table 1 - supplementary material). Finally, outliers were removed by applying 1st and 99th percentile cutoffs sequentially: first to individual CBC parameters, and subsequently to the derived indices. This two-stage trimming ensured that reference intervals remained unaffected by extreme values. Thus, the sample of the present study consisted of 2230 participants (Figure 1).

**Table 1:** Demographic and socioeconomic characteristics of the study participants. N = 2230.

Characteristic	Frequency n (%)
**Study Centre**	
São Paulo	744 (34.71)
Minas Gerais	482 (21.61)
Bahia	324 (14.53)
Rio de Janeiro	276 (12.38)
Rio Grande do Sul	202 (09.06)
Espírito Santo	172 (07.71)
**Sex**	
Male	1211 (54.30)
Female	1019 (45.70)
**Age (years)**	
35–59	2052 (92.02)
≥ 60	178 (07.98)
**Self-reported skin colour**	
White	1172 (53.15)
Mixed	675 (30.61)
Black	276 (12.52)
aOthers	82 (3.72)
**Schooling (years)**	
< 11	173 (7.75)
11–14	752 (33.72)
≥ 15	1305 (58.52)

a includes native indigenous people (n = 20; 0.94%), Asian descendants (n = 59; 2.78%) and absent (n = 24).

**Figure 1:**
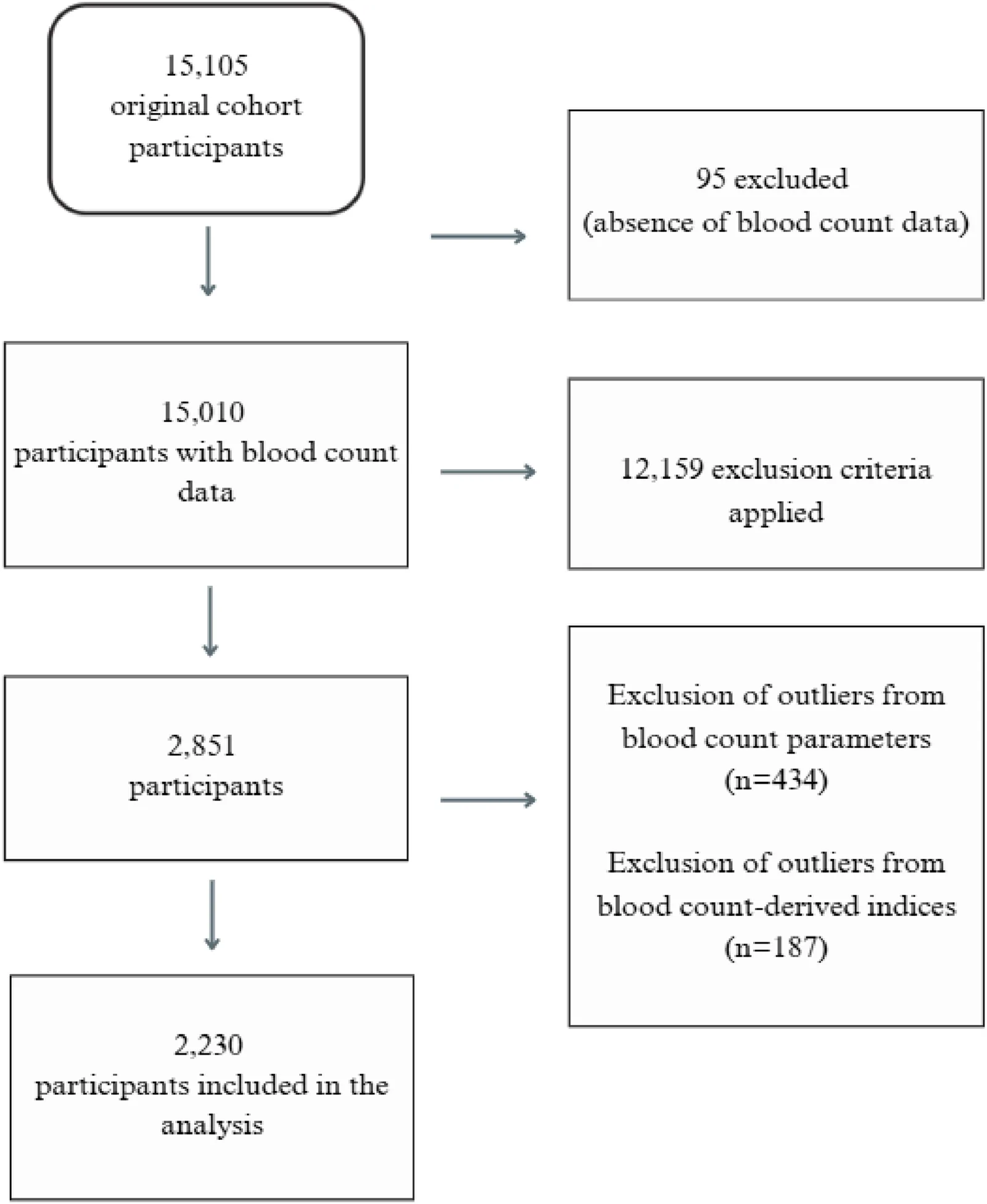
Flowchart of the study population.

*Values below the 1st percentile and above the 99th percentile were excluded

### Biological sample and calculation of indices derived from the complete blood count

Venous blood was collected in the morning following a 12-hour overnight fast, in accordance with Clinical and Laboratory Standards Institute (CLSI) venipuncture protocols. Samples were maintained at room temperature and processed within two hours of collection for CBC analysis [21]. Phlebotomy was performed using an evacuated tube system, with tourniquet application limited to less than one minute to minimize hemoconcentration. Blood was drawn into sample tubes containing tripotassium EDTA, each labeled with a unique participant barcode to ensure specimen tracking.

Due to technical considerations, CBC analyses were performed locally using automated hematology analyzers. All participating laboratories maintained internal and external quality control standards and participated in accredited proficiency programs (National Quality Control Program, Laboratory Testing Proficiency Program, and College of American Pathologists Accreditation Program) [22].

All cell counts were expressed as absolute values (x10^9^/L). The indices were calculated using the following formulas:

• NLR = Neutrophils / Lymphocytes
• PLR = Platelets / Lymphocytes
• LMR = Lymphocytes / Monocytes
• MLR = Monocytes / Lymphocytes
• dNLR = Neutrophils / (Total Leukocytes - Neutrophils)
• SIRI = (Neutrophils x Monocytes) / Lymphocytes
• AISI = (Neutrophils x Monocytes x Platelets) / Lymphocytes
• SII = (Platelets x Neutrophils) / Lymphocytes

The formulas used for dNLR, SIRI, AISI, and SII were based on previously published studies describing these complete blood count-derived inflammatory indices [[9], [10], [11]].

### Statistical analysis

The Kolmogorov-Smirnov test was used to assess the normality of the indices derived from the CBC. A nonparametric method was used to determine the reference intervals, calculated as the interval from the 2.5th percentile to the 97.5th percentile of the distribution of the parameters. The Mann-Whitney U test was used to verify differences between subgroups defined by sex and age. Subsequently, the need to recommend a specific reference interval for the age and sex groups was assessed using the Harris-Boyd method. Using this approach, the z scores of the means and standard deviations (SDs) of the hematologic parameters and indices were calculated and compared with a critical value (z*). Reference intervals separated by age group and sex were recommended if at least one of the following conditions was met [21]:

1) The calculated z exceeded the critical value z*;
2) There were differences between the SDs of the hematologic parameters of each subgroup and if the ratio of the maximum to minimum SD exceeded 1.5, or if the variance ratio [largest SD / (largest SD - smallest SD)] was <3.0.

A p-value <0.05 was considered statistically significant. Statistical analysis was performed using the STATA 14.0 statistical package (Stata Corporation, College Station, United States).

## Results

Of the 2230 participants, the majority belonged to the São Paulo study center (34.7 %), were men (54.3%), aged 35–59 years (92.0%), self-declared white (53.15 %), and had completed higher education (58.5%) (Table 1).

Histograms showing the distribution of the indices derived from the CBC are presented in Figure 2.

**Figure 2:**
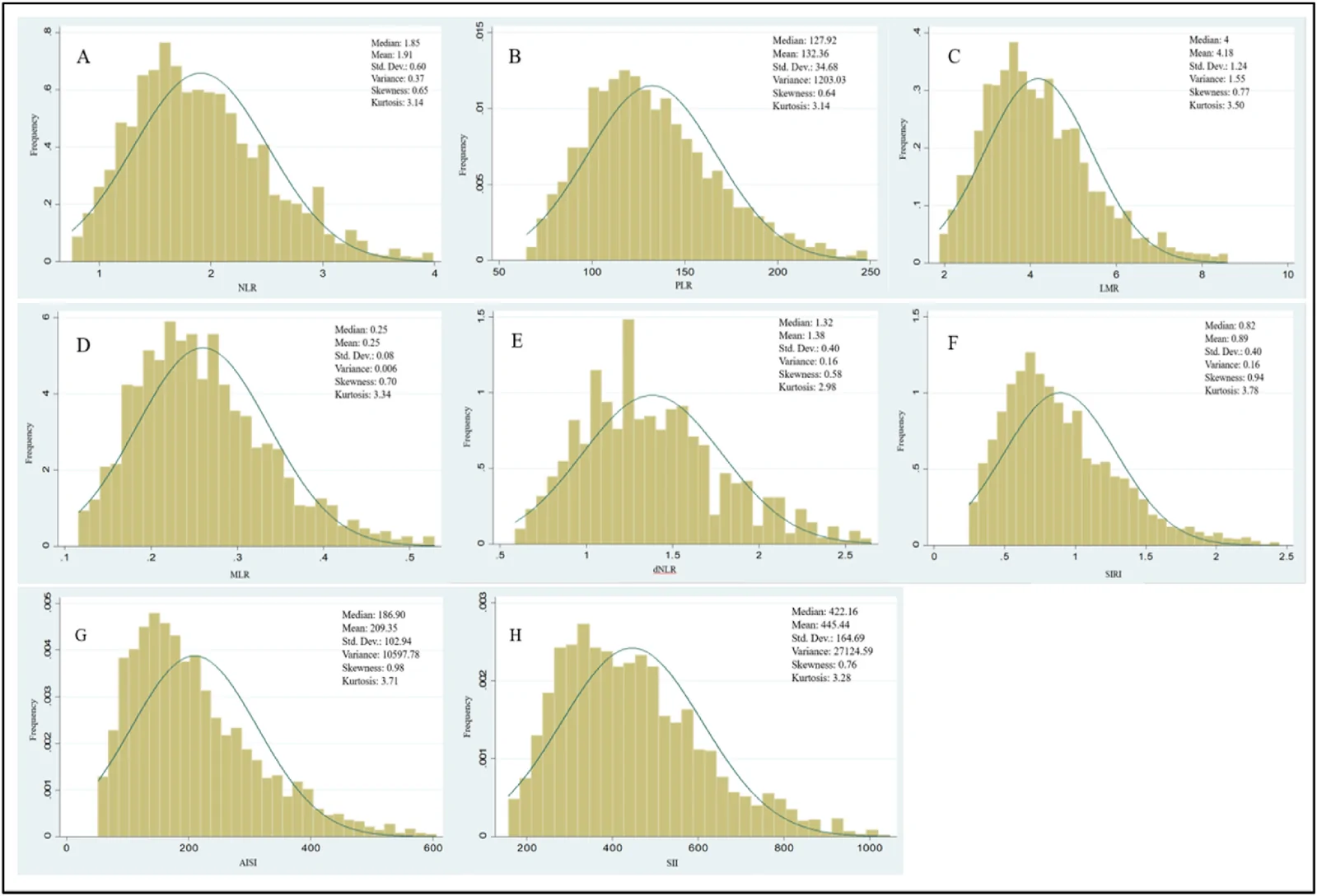
Distribution of inflammatory hematologic indices in the sample.

Panel A: Neutrophil-lymphocyte ratio; Panel B: platelet-lymphocyte ratio; Panel C: lymphocyte-monocyte ratio; Panel D: monocyte-lymphocyte ratio; Panel E: derived neutrophil-lymphocyte ratio; Panel F: systemic inflammation response index; Panel G: aggregate index of systemic inflammation; Panel H: systemic immune-inflammation index.

Regarding sex, there was a statistically significant difference for most of the indices derived from the CBC, except for the NLR. These results regarding sex showed that the median values of the MLR and SIRI indices were higher in men, and the PLR, LMR, dNLR, and SII in women. Regarding age, there was a statistically significant difference only for the SII index, in which the median was higher in the 35- to 59-year age group (Table 2).

**Table 2:** Assessment of indices derived from the complete blood count in relation to sex and age group (n = 2230).

	N total (n = 2230)	Sex			Age		
		Male (n = 1211)	Female (n = 1019)	P-value	35 to 59 years (n = 2052)	≥ 60 years (n = 178)	P-value
NLR	1.82 (1.46–2.28)	1.81 (1.46–2.27)	1.84 (1.45–2.29)	0.427	1.82 (1.46- 2.28)	1.84 (1.47- 2.23)	0.642
PLR	127.92 (106.72–226.47)	121.7 (102.56–145.39)	135.87 (112.47–161.76)	< 0.001	127.88 (106.50–153.43)	127.98 (109.37–149.00)	0.919
LMR	4 (3.27–4.90)	3.85 (3.13–4.69)	4.20 (3.47–5.17)	< 0.001	4.00 (3.28- 4.91)	3.79 (3.19- 4.83)	0.087
MLR	0.25 (0.20–0.30)	0.26 (0.21–0.32)	0.24 (0.19–0.28)	< 0.001	0.25 (0.20–0.30)	0.26 (0.21–0.32)	0.087
dNLR	1.32 (1.08–1.63)	1.31 (1.08–1.58)	1.36 (1.08–1.64)	0.005	1.32 (1.08- 1.63)	1.32 (1.04–1.56)	0.179
SIRI	0.82 (0.60–1.12)	0.84 (0.62–1.16)	0.78 (0.59–1.07)	0.001	0.82 (0.60–1.12)	0.80 (0.61- 1.07)	0.595
AISI	186.90 (132.36–266.33)	181.95 (129.04–263.35)	193.37 (137.73–271.47)	0.024	187.38 (133.38–269.20)	173.93 (125.90- 247.91)	0.095
SII	422.16 (319.16–546.93)	395.50 (304.39–506.55)	455.18 (339.74–578.28)	< 0.001	423.75 (319.75–549.21)	395.72 (309.72–500.33)	0.043

^a^P-value obtained by Mann Whitney test.

^b^paired *t*-test; Data are presented as median (25–75 percentile) or mean ± standard deviation.

NLR: Neutrophil-lymphocyte ratio; PLR: platelet-lymphocyte ratio; LMR: lymphocyte-monocyte ratio; MLR: monocyte-lymphocyte ratio; dNLR: derived neutrophil-lymphocyte ratio; SIRI: systemic inflammation response index; AISI: aggregate index of systemic inflammation; SII: systemic immune-inflammation index.

The z statistic was calculated for the indices that presented significant differences in regards to sex and age. In the analysis by age and sex, no index presented any of the conditions of the Harris-Boyd method for categorizing reference intervals. Therefore, we chose not to present categorized reference intervals for the indices studied (Table 3).

**Table 3:** Reference intervals for indices derived from the complete blood count (total n = 2230).

Index	Median	Reference Interval	
		2.5 Percentile	97.5 Percentile
NLR	1.82	0.95	3.28
PLR	127.92	77.25	213.27
LMR	4.00	2.27	7.17
MLR	0.25	0.14	0.44
dNLR	1.32	0.72	2.33
SIRI	0.82	0.33	1.89
AISI	186.90	69.85	460.01
SII	422.16	201.64	830.02

NLR: Neutrophil-lymphocyte ratio; PLR: platelet-lymphocyte ratio; LMR: lymphocyte-monocyte ratio; MLR: monocyte-lymphocyte ratio; dNLR: derived neutrophil-lymphocyte ratio; SIRI: systemic inflammation response index; AISI: aggregate index of systemic inflammation; SII: systemic immune-inflammation index.

## Discussion

This study calculated the reference intervals of complete blood count-derived indices obtained from a large cohort of individuals aged 35 to 74 years. Although this cohort does not fully capture the heterogeneity of the Brazilian population, these findings contribute valuable data toward understanding the distribution of these indices and represent an important step in their potential use as diagnostic and prognostic tools, which should be further validated in more diverse populations.

The clinical relevance of complete blood count-derived indices is supported by the different components of the immune-inflammatory response represented by each index. NLR reflects the balance between neutrophil-mediated innate inflammation and lymphocyte-mediated adaptive immunity, whereas MLR reflects the relationship between monocyte-driven inflammatory activity and immune competence. SIRI integrates neutrophils, monocytes, and lymphocytes, providing a broader estimate of systemic inflammatory burden. Platelet-based indices, including PLR, SII, and AISI, additionally incorporate the interaction between inflammation, thrombosis, and vascular injury. These biological characteristics help explain the growing use of complete blood count-derived indices as markers of systemic immune-inflammatory activation in infectious, cardiovascular, neoplastic, and other chronic inflammatory conditions. Li et al. recently emphasized that NLR, MLR, and SIRI capture distinct dimensions of inflammatory and immune dysregulation, supporting their interpretation beyond isolated leukocyte counts [23]. In addition, recent NHANES analyses have shown that indices such as NLR, PLR, SII, and SIRI are associated with adverse clinical outcomes and mortality in chronic inflammatory diseases, highlighting their potential as accessible and low-cost biomarkers for clinical and epidemiological applications [24]. Currently, there is a wide and inconsistent range of cut-off points for these indices reported in the literature [25], but it is essential to extract these data in an appropriate context to properly evaluate the reference intervals. Many retrospective studies have proposed "high risk" cut-off points for some indices based on Kaplan-Meier curves and multivariate Cox regression analyses, however, some are not representative due to the small amount of data and incomplete coverage area [16].

Some studies that also used the recommendations proposed by the CLSI pointed out the need to stratify the indices by sex and/or age [16,26,27] (Table 4). However, in the present study, this stratification was not necessary since none of the conditions of the Harris-Boyd method were met. This study found a median NLR index of 1.82 for Brazilian men and women. However, Chinese studies showed that the median values for NLR were lower with an average of 1.55 for men and 1.59 for women [16] and 1.77 for the general population [26]. In a Rotterdam study carried out with a Dutch population, the average was 1.76 for the general population [27]. Ezequiel et al. found an average of 1.92 for the Argentine population, which is higher than that found in the current study [28] (Table 4).

**Table 4:** Reference ranges for indices derived from the complete blood counts of different studies.

Study	Study population	Country of origin	Reference Interval							
			NLR	PLR	LMR	SII	MLR	dNLR	SIRI	AISI
ELSA-Brasil, 2025	2230 healthy individuals, aged 35 to 74 years (1211 men; 1019 women)	Brazil	1.82 (0.95- 3.28)	127.92 (77.25- 213.27)	4.00 (2.27–7.17)	422.16 (201.64- 830.02)	0.25 (0.14–0.44)	1.32 (0.72–2.33)	0.82 (0.33–1.89)	186.90 (69.85–460.01)
Wang et al., 2021 [13]	404,272 healthy Chinese adults, aged 18 to 112 years (205,592 men; 198,680 women)	China	Men: 1.5 (1.25–1.93); Women: 1.59 (1.27–2.0)	Men: 102.0 (84.7–123.24); Women: 116.57 (96.28–141.05)	Men: 5.14(4.22–6.26); Women: 5.50(4.49–6.88)	**-**				
Ezequiel et al., 2021 [23]	71,873 non -hospitalized individuals aged 16 to 103 years (24,316 men; 47,557 women)	Argentina	1.92 (1.46- 2.58)	-	-	-				
Luo et al., 2019 [21]	5969 healthy Chinese individuals aged 18 to 79 years (3047 men; 2.722 women)	China	1.77 (0.88–4.0)	99 (48–197)	5.27 (2.63–9.90)	334 (142–804)				
Fest et al., 2018 [22]	8711 healthy individuals aged 45 years and over (3733 men; 4978 women)	Netherlands	1.76 (0.83–3.92)	120 (61–239)	-	459 (189–1168)				

NLR: Neutrophil-lymphocyte ratio; PLR: platelet-lymphocyte ratio; LMR: lymphocyte-monocyte ratio; MLR: monocyte-lymphocyte ratio; dNLR: derived neutrophil-lymphocyte ratio; SIRI: systemic inflammation response index; AISI: aggregate index of systemic inflammation; SII: systemic immune-inflammation index.

The mean PLR (127.92) was higher than the mean observed in other studies: 102.0 for men and 116.57 for women [16], and 99.0 and 120.0 for the general population [26,27]. Chinese studies showed that the mean LMR values were higher than those found in this study. In studies with the Chinese population, the mean varied from 5.14 for men and 5.50 for women [16] and 5.27 for the general population [26], while in this study the mean was 4.0 (Table 4).

Regarding the SII index, this study found an average of 422.16, higher than the average of 334.0 found in the Chinese study by Luo et al. [26], and lower than the average of 450.0 obtained by Fest et al. [25] (Table 4). The different values found in the studies, as shown in Table 4, can be justified by the known hematological differences between the populations studied (Caucasians, Asians and Latinos) and by the differences attributed to environmental, cultural, and dietary factors in the regions of the studies [29], as well as the sample size and pre-analytical and analytical variables used in the studies. There is a scarcity in the literature of studies that address reference intervals and the MLR, dNLR, SIRI and AISI indices.

Establishing reference intervals for laboratory tests is a methodical, rigorous, and quite challenging process, however, each population should have its own reference values. Care must be taken to follow methodological rigor, adequate sample sizes and characteristics, standardization and control during collection, processing, transportation, and analysis. Due to the difficulty in conducting their own studies, some developing countries end up using parameters from research conducted in developed countries as a reference, which may not accurately represent the population in question [30].

It is worth noting that this study only includes healthy adults (35 to 74 years old) and does not analyze data on adolescents, children, and other specific groups, such as pregnant women. Therefore, the study is limited to the ELSA-Brasil population, which makes the results not representative of the entire Brazilian population. However, adults from three regions of Brazil participated, which adds power to the diversity of the sample. The large sample size constitutes a primary strength of this study, enabling robust estimation of representative reference intervals for hematological indices in adults aged 35–74 years.

## Final considerations

Establishing population-specific reference intervals provides more reliable baseline estimates of participant health status, ultimately advancing clinical decision-making and disease prevention strategies. By establishing these indices in the ELSA-Brasil baseline sample may stimulate studies aimed at evaluating these indices in different clinical conditions, especially chronic non-communicable diseases in the Brazilian population and discover whether these indices can be predictors of mortality when above the reference interval value.

## Ethics and consent

The procedures followed were in accordance with the ethical standards of the committee responsible for human experimentation and with the 1975 Declaration of Helsinki, as revised in 1983. ELSA-Brasil was approved by the Research Ethics Committees of the participating institutions and by the National Commission for Research Ethics (CAAE 0016.1.198.000–06) /(CONEP 976/2006) of the Ministry of Health. All study participants signed the Informed Consent Form.

## Conflicts of interest

The authors declare that there are no conflicts of interest. The ELSA-Brasil baseline study was supported by the Brazilian Ministry of Health (DECIT – Science and Technology Department) and the Brazilian Ministry of Science and Technology (FINEP, Financiadora de Estudos e Projetos and CNPq, National Research Council), grants 01 06 0010.00 RS, 01 06 0212.00 BA, 01 06 0300.00 ES, 01 06 0278.00 MG, 01 06 0115.00SP, 01 06 0071.00 RJ. Rios D.R.A. and Barreto S.M. are CNPq research fellow of the National Research Council (CNPq, grants n° 311,117/2025–2 and 300,159/99–4, respectively) and Barreto S.M. is supported by a research grant (Pesquisador Mineiro) from FAPEMIG. This study was financed in part by the Coordenação de Aperfeiçoamento de Pessoal de Nível Superior - Brasil (CAPES) - Finance Code 001.

## Data Availability

The data that support the findings of this study are available from the corresponding author upon reasonable request

## Appendix Supplementary materials

Image, application 1
