# Supplementary material for: Reference intervals for indices derived from the complete blood count in the Longitudinal Study of Adult Health (ELSA-Brasil)
Source: Hematol Transfus Cell Ther. 2026 Oct 15;49(1):108552. doi: 10.1016/j.htct.2026.108552 (PMC13691253; doi:10.1016/j.htct.2026.108552)
Supplement: Supplementary file 1 [file mmc1.docx]

**Supplementary Material**

**Table 1: Exclusion criteria for participants (N = 15,105)**

| **Exclusion criteria (n=15,105)** | **Excluded** | **Included** |
| --- | --- | --- |
| Missing blood count data | 95 | 15,010 |
| Health problems in the last 12 hours | 1,104 | 13,911 |
| C-reactive protein >10 | 563 | 13,348 |
| Taking any regular medication | 7,733 | 5,610 |
| State of health (fair, poor, very poor) | 690 | 4,920 |
| Self-reported medical diagnosis of diabetes mellitus | 54 | 4,866 |
| Self-reported medical diagnosis of hypertension | 401 | 4,465 |
| Self-reported medical diagnosis of cardiovascular disease^a^ | 96 | 4,369 |
| Self-reported medical diagnosis of thrombosis or pulmonary embolism | 36 | 4,333 |
| Self-reported medical diagnosis of liver disease^b^ | 336 | 3,997 |
| Cancer | 76 | 3,921 |
| Current smoke^c^ | 536 | 3,385 |
| Body mass index (BMI) ≥30 kg/m^2^ | 484 | 2,901 |
| Glomerular filtration rate (GFR) <60 mL/ min/1.72 m^3^ | 50 | 2,851 |
| Exclusion of extreme hematological parameters (outliers)^d^ | 621 | 2,143 |
| **Total** | **2,230** | |

^a^Acute myocardial infarction, angina, congestive heart failure, stroke and myocardial revascularization; ^b^cirrhosis or hepatitis; ^c^participants who declared that they had smoked at least one hundred cigarettes over the course of their lives and were still smoking; ^d^outliers excluded after evaluation of the 1^st^ and 99^th^ percentiles
